# Performance of the BD Veritor system SARS-CoV-2 antigen test among asymptomatic collegiate athletes: A diagnostic accuracy study

**DOI:** 10.1016/j.rcsop.2021.100047

**Published:** 2021-07-08

**Authors:** Casey M. Kepczynski, Jaelin A. Genigeski, Daniel P. de Regnier, Emmanuel D. Jadhav, Minji Sohn, Michael E. Klepser

**Affiliations:** Ferris State University College of Pharmacy, 1201 S. State St., Big Rapids, MI 49307, United States.

**Keywords:** BD Veritor system, Antigen-test, PCR-test, Cost-analysis, SARS-CoV-2

## Abstract

**Background:**

COVID-19 testing strategies and determining the accuracy of tests is crucial for the prevention of disease in asymptomatic communities.

**Objective:**

To determine the positive predictive value for the BD Veritor System for rapid detection of SARS-CoV-2 (BD Veritor System) among asymptomatic athletes and athletic staff in a University setting. Secondarily, a cost savings analysis was conducted to evaluate the benefits of a rapid antigen testing strategy over a universal PCR-based strategy.

**Methods:**

Asymptomatic athletic personnel at Ferris State University tested using the BD Veritor System from November 4, 2020-February 15, 2021. Individuals whose antigen test was positive immediately had specimen collected for confirmatory PCR testing. These results were used to determine the positive predictive value (PPV) for the BD Veritor System. A cost-savings analysis was conducted from the University's perspective to determine the value of this rapid antigen testing strategy over a universal PCR-based strategy.

**Results:**

A total of 3352 antigen tests were performed on 359 individuals during the study period. During this period, 21 positive antigen tests were obtained of which 5 individuals had a positive reflex PCR result. The calculated PPV of the BD Veritor System among asymptomatic individuals was 25%. According to the mandated athletics testing schedule, the University spent $67,475.76 on BD Veritor System tests and $1785 on confirmatory PCR tests. In contrast, if a solely PCR-based approach had been continued, the same testing strategy would have cost the University $284,920. By employing a 2-tiered testing strategy with the BD Veritor System with reflex PCR testing, the University realized a cost savings of $215,659.24 during the 3-month period.

**Conclusions:**

Despite sub-optimal PPV associated with the BD Veritor System among asymptomatic athletes, the University was able to effectively use an antigen-based testing program to comply with collegiate testing requirements and realize $215,659.24 cost savings per quarter over a PCR-based strategy.

## Highlights

• BD Veritor System analyzer is $330.00 for each analyzer (the University purchased 10 analyzers).
• Each BD Veritor analyzer is expected to perform 2500 tests.
• The University had a capital outlay of $3,300.00 for 10 analyzers. If 10 analyzers run 25,000 tests, they contributed $0.13 to the cost of each test.

## 1 Introduction

As of February 1, 2021, more than 26 million cases of COVID-19 have been reported in the US.^1^ Controlling the spread of SARS-CoV-2 has been hindered by the fact that 30%–50% of those infected may be asymptomatic.^1^ Despite not exhibiting symptoms, these individuals have been implicated in being a source of viral spread. Early in the course of the pandemic, antigen and PCR-based tests were developed to identify SARS-CoV-2 among those with symptoms in an effort to isolate these infected individuals quickly. This allowed us to optimize the use of limited resources and protect healthcare workers and vulnerable populations. As the pandemic continued, various testing resources became more widely available, and the focus of testing began to shift toward identifying asymptomatic carriers of the virus. The rationale behind this change was to slow the spread of SARS-CoV-2 and allow some cohorts of the population, such as athletes, to resume daily activities safely. Athletic programs began requiring smooth testing techniques to become implemented in order to recommence sporting events safely. Identifying safety measures for college athletes, in particular, became important to identify the potential for asymptomatic carriers around team members and prevent the spread of disease to athletics and Universities as a whole. The strategy at the University in this study requires that individuals be tested frequently, obtain results rapidly, and be run at the point of testing. This is important to determine in real-time to identify team members quickly before the potential of spreading. Additionally, because of the number of tests that must be run to achieve the goals of asymptomatic testing, the ideal test is needed to be relatively inexpensive. The University began to evaluate test platforms for asymptomatic screening in August 2020 as students and student-athletes came back to campus.

Two SARS-CoV-2 testing platforms dominate the testing market, those that utilize real-time reverse-transcriptase polymerase chain reaction (RT-PCR) and those that detect SARS-CoV-2 nucleocapsid antigen. RT-PCR tests for various genes specific to SARS-CoV-2.^2^ When performed in a high complexity laboratory setting, RT-PCR tests demonstrate high sensitivity and specificity. Despite excellent performance, numerous shortcomings were found when valuing the high complexity PCR-based tests for asymptomatic screening programs. First, there is a considerable delay in obtaining test results, typically 48 h or more. Second, when these tests were performed, they were done at an outside laboratory, costing $75–$100 per test. Lastly, PCR tests are not able to discriminate between viable viruses associated with active infection and genetic fragments that persist from the previous infection. Conversely, SARS-CoV-2 antigen tests provide results within minutes, are relatively inexpensive, and are less likely to detect non-viable viruses. Unfortunately, antigen tests were authorized for use in individuals with symptoms or in whom the virus's presence was suspected. Under conditions like this, when the pre-test probability of disease is high, antigen tests have relatively good diagnostic performance. Unfortunately, since the pre-test probability of infection with SARS-CoV-2 is much lower among asymptomatic populations (such as the collegiate athletic population in question), it was anticipated that the diagnostic performance of the antigen tests would be much lower.

Sensitivity and specificity reflect the intrinsic performance characteristics of a test compared to a gold standard. These characteristics are not influenced by the pre-test probability of a disease of interest. These values are easy to determine since they only require specimens that contain and do not contain the agent of interest. The FDA review process allows for these characteristics to be determined on spiked samples. As a result, sensitivity and specificity provide insight into the performance of the test compared to the gold standard; however, they do not provide complete insight into the clinical utility of the test. In this case, since we lack a gold standard for SARS-CoV-2 testing, sensitivity can be referred to as positive percent agreement (PPA) and specificity as negative percent agreement (NPA), respectively. PPA and NPA are more accurate representations of results when a true reference standard is not present, and therefore, can be evaluated to PPA/NPA results of comparable testing platforms (other antigen-based SARS-CoV-2 tests). To gain a perspective on a test's clinical performance, the positive predictive value (PPV) and negative predictive value (NPV) are used. These values examine the test's predictive values considering the pre-test probability of disease in a population.^3^ For example, if a test with high specificity is used in a symptomatic population when the pre-test probability is high, then the number of false-positive results will be low and the PPV will be high. On the other hand, if the same test is run in an asymptomatic population, the pre-test probability is low. Therefore, the rate of false-positive results will be higher; PPV will be lower. The same type of relationship can be expected with NPV, respectively. For the antigen-based tests, it was noted that although the PPV among symptomatic individuals would likely be high, this was because this was the population they were authorized for use. However, since the tests had not been authorized for use among asymptomatic individuals, confidence was low in the PPV of the athletics population.

As the University weighed the variables associated with various testing platforms, it was determined that using an antigen-based testing strategy would meet the needs for asymptomatic testing better than a PCR-based approach. Among this population, we assume the NPV of the available tests would be satisfactory; however, PPV in asymptomatic individuals would be low, resulting in a high percentage of false positives. As a result, all asymptomatic individuals with a positive antigen test would require confirmation using a laboratory-based PCR test. Owing to the product availability, the University elected to begin using the BD Veritor System for Rapid Detection of SARS-CoV-2 antigen test. As performance data became available with other antigen-based tests reviewed, it would be prudent to assess the performance of the BD Veritor System antigen test and perform a cost analysis to determine the budgetary impact of or decisions on the University.

## 2 Methods

This was an observational, prospective, diagnostic accuracy study of the BD Veritor System for Rapid Detection of SARS-CoV-2 antigen test among asymptomatic athletic personnel over the age of 18. The study period was from November 4, 2020, to February 15, 2021. This project was classified as exempt by the Ferris State University IRB. Financial support was given via Ferris State University to provide adequate supplies for testing and protective equipment; the University was not involved in the study design, collection of data, or interpretation of data. The University's athletic programs include about 460 athletes and 15 head coaches. Eligibility comprised of all members of the Ferris State University athletic program (i.e., athletes, coaches, trainers) who were required to submit to regular testing according to the National Collegiate Athletic Association (NCAA), Michigan Department of Health and Human Services (MDHHS), and Great Lakes Intercollegiate Athletic Conference (GLIAC) guidance. Based on pooling the recommendations of college athletic programs, the most stringent of testing requirements was used at the University from MDHHS guidelines listed on January 4th, 2021, prior to vaccine availability: all athletic personnel was to be tested 6 times weekly if team members and personnel are practicing and playing without the use of a face mask.^4^ Based on MDHHS guidelines, a confirmatory negative result had to be tested within a day of unmasked play. All individuals must have completed a University symptom checker and attest that they were symptom-free prior to testing. Individuals typically used a single swab to self-collect a nasal specimen from both nostrils; staff members were able to assist with nasal collection if individuals could not themselves. University personnel staffing the testing clinics were trained to observe and assist with specimen collection and perform the test. All tests were performed in accordance with the manufacturer's user guide submitted for Emergency Use Authorization.^5^ The article itself was followed based on “Standards for Reporting Diagnostic accuracy studies”; STARD guidelines for the procedures taken place. In consideration of competing interests, there are none to declare at this time.

Data on SARS-CoV-2 activity over the study period were reported to District 10 Health Department, which includes Mecosta County and Ferris State University. Individuals for whom a positive test was obtained from the BD Veritor System Rapid Detection of SARS-CoV-2 antigen test were contacted for test confirmation. Confirmatory tests were run at GeneMarkers Laboratory (Kalamazoo, MI) using the TaqPath COVID-19 Combo Kit (Thermo Fisher Scientific, Carlsbad, CA).

### 2.1 Statistical analysis

Test result data were described using descriptive statistics. Additionally, the rate of test positivity and positive predictive value (PPV) of the BD Veritor System for Rapid Detection of SARS-CoV-2 antigen test were calculated. Performance of the BD Veritor test was also stratified by month to examine fluctuation of PPV as community prevalence of disease activity changed.

A cost avoidance analysis was conducted to examine the financial impact of the employed testing strategy from a University perspective versus a complete PCR-based testing strategy. Since the personnel and personal protective equipment costs were similar to the two testing strategies, the focus was solely on the cost of each of the tests.

## 3 Results

**Fig. 1:**
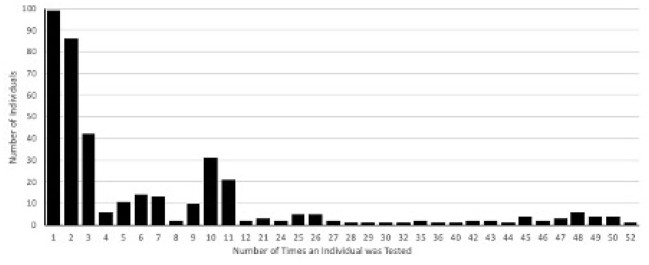
Testing frequency among athletic community.

**Fig. 2:**
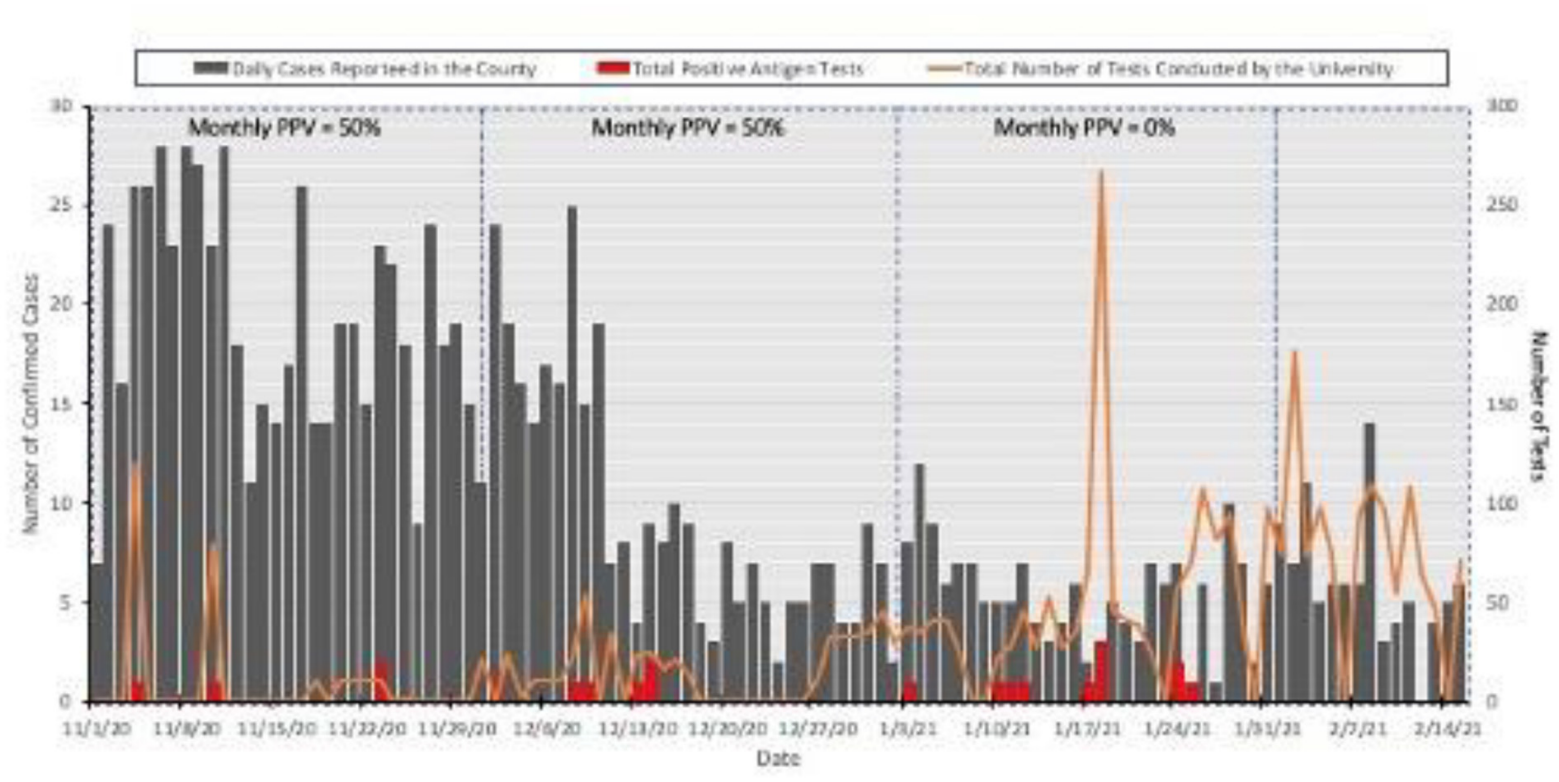
Summary of daily cases reported to the County and University asymptomatic testing among athletics.

During the study period, 3352 tests were performed on 359 individuals from 15 sports teams (Table 1). Individuals were tested a median of 3 times (range 1–52, Fig. 1). A total of 21 positive antigen tests were reported from 18 individuals. The rate of antigen test positivity was 0.63% over the study period. Of the 21 positive antigen tests, 5 were confirmed as positive by PCR testing. This translated into a PPV of 23.8% for the study interval. Monthly PPV's were 50%, 50%, 0% for November, December, and January. The month of January had a 0% PPV, given that the confirmatory PCR tests were negative. February was non-calculable due to no positive antigen tests that month (Table 1). Over the study period, 1150 confirmed cases of COVID-19 were identified in Mecosta County.^6^ The number of new daily cases per 100,000 in the county ranged from 8.2 to 63.8, and the daily test positivity rate ranged from 3.8% to 14.1%^7^ Fig. 2 depicts the daily cases of SARS-CoV-2 in Mecosta County from November 5, 2020-February 15, 2021, and data from the University antigen testing program.

**Table 1:** Summary of asymptomatic test data.

Sport Group	Number of Individuals	Number of Tests Performed	Number of Positive Antigen Tests	Number of Confirmatory PCR Tests	Number of Positive PCR Tests
Football	133	266	2	2	1
Men's Basketball	18	531	6	6	3
Men's Cross Country/Track & Field	17	163	0	0	0
Men's Golf	8	13	0	0	0
Men's Ice Hockey	28	980	10	10	1
Men's Tennis	5	22	0	0	0
Men's Track & Field	15	106	0	0	0
Softball	23	47	0	0	0
Volleyball	16	152	0	0	0
Women's Basketball	13	578	0	0	0
Women's Cross Country/Track & Field	17	132	3	3	0
Women's Golf	10	17	0	0	0
Women's Soccer	34	182	0	0	0
Women's Tennis	10	85	0	0	0
Women's Track & Field	11	76	0	0	0
Sports Medicine Aide	1	2	0	0	0
**Totals**	**359**	**3352**	**21**	**21**	**5**

Some outliers remained as a part of the testing strategy. Two individuals tested positive multiple times with the antigen test. Each time the result was determined to be a false positive upon confirmatory testing with PCR. One athlete had been diagnosed with COVID-19 in December 2020 and was exempt from testing for 90-days. Upon resumption of testing, the person produced 3 consecutive positive antigen tests that were disproved by PCR. Another individual had 2 consecutive positive antigen tests that were determined to be false positives by PCR. This individual did not have a history of infection with SARS-CoV-2 and has subsequently been tested with the antigen test 8 times, all of which have been negative.

If the university had used a strictly PCR-based testing strategy as had originally been implemented, the total costs to conduct the 3352 tests would have been $284,920.00 (Table 2). In contrast, the total cost associated with performing the antigen tests was $69,260.76. During the roughly 3.5-month testing timeframe, the University realized an approximate savings of $215,659.24.

**Table 2:** Cost avoidance for the tested population (*n* = 3352).

Item	Unit Cost per Test	Units Consumed for Testing	Cost
PCR-Based Strategy Alone			
PCR Test	$85.00	3352	$284,920.00
Total			$284,920.00
BD Veritor System Antigen Test with PCR Confirmation of Positive Results			
PCR Test	$85.00	21	$1785.00
BD Veritor Antigen Test	$20.00	3352	$67,040.00
BD Veritor System	$0.13	3352	$435.76
**Total**			**$69,260.76**
**Cost Savings**			**$215,659.24**

## 4 Discussion

In the early months of the pandemic, the ability to accurately detect SARS-CoV-2 among individuals was paramount. The main goal was to identify infected people early on and quarantine them as quickly as possible. In this environment, the use of tests with high sensitivities and specificities was essential. Under these conditions, individuals would likely only need testing when their symptoms or exposure history dictated. In these scenarios, central-laboratory-based PCR-based tests were valuable because the willingness to trade improved accuracy for a longer time to get results. As the pandemic moved into the fall, many US colleges and universities and other settings such as nursing homes, correctional facilities, and detention facilities began to use antigen-based testing on asymptomatic individuals to facilitate early case identification.^8^ Universities then sought to allow athletics programs to resume some level of activity. To accomplish this, the strategy and goal of testing shifted to ensure that the individuals associated with the athletics programs were not infected with SARS-CoV-2. To meet this goal, the need was to develop a strategy that allowed testing of asymptomatic individuals multiple times a week and provide results virtually in real-time. This strategy would allow athletes with negative test results to be cleared for participation. Under these parameters, the ideal test for athletics testing would be inexpensive, provide results in minutes, and allow the University to be confident that individuals testing negative were truly negative. Additionally, it was recognized that false-positive tests would be encountered, so a plan for reflex testing of positive tests would need to be established. Antigen-based testing platforms were identified as good candidates for this testing strategy.

As a group, the antigen-based tests available for SARS-CoV-2 are reported to have positive percent agreement values (PPA) ranging from 85%–95% and negative percent agreement values (NPA) of >99% used in place of specificity when an accepted gold-standard test does not exist for comparison.^9^ Therefore, in an asymptomatic population like the University's athletes, coaches, and trainers, where the pre-test probability of an individual being infected with SARS-CoV-2 is low, antigen tests should be expected to provide good NPV. However, even with high NPA's/specificities, false-positive results are to be expected. This performance pattern has been reported following testing in asymptomatic groups with the Abbott BinaxNOW Rapid Antigen Test for SARS-CoV-2 (Abbott, Abbott Diagnostics) and the Sofia SARS Antigen Fluorescent Immunoassay (Sofia, Quidel Corporation).^8^^,^^10^ Among a cohort of 2592 asymptomatic individuals, the PPV and NPV for the Abbott test were calculated to be 91.7% and 96.9%, respectively.^10^ Another study of antigen-based testing for asymptomatic individuals was done at two universities in Wisconsin using the Sofia SARS detection test, with a similar population to this study of University students. Values of testing done at both universities concluded with a PPV of 33.3% and an NPV of 89.9%.^8^ The authors of the study concluded that owing to low costs and rapid turnaround times, antigen tests may be appropriate for use for screening for SARS-CoV-2 among asymptomatic individuals as long as a mechanism was in place to confirm positive results. In this study with the BD Veritor System, a comparably low PPV was noted, 23.8%, among asymptomatic individuals. Although there was no access to disease prevalence for Mecosta County in this timeframe, the test positivity was reported to range from 3.8% to 14.1%^6.^ Using these values as rough surrogates for disease prevalence, data from the manufacturer's package insert suggest that users could expect a PPV of 50% at a disease prevalence of 0.6% and a PPV of 92% at a prevalence of 6.0%.^11^ The observed PPVs appeared to be lower than expected given the reported County test positivity rate. Fluctuations were observed in calculated PPV between 0%–50% in the program. The higher PPVs noted were in November and December when the County test positivity rates were highest. These findings highlight the fact that community disease prevalence should be monitored to assist with interpreting test results obtained when testing asymptomatic individuals.

Neither the Abbott nor Sofia study examined the cost impact of using an antigen test followed by confirmatory testing of positive results compared to universal testing with a PCR-based test. At this specific institution, the capability of running clinical SARS-CoV-2 specimens on campus was not available. As a result, partnering with off-campus laboratories was necessary to test these samples. The cost for PCR-based tests has ranged from $75–$100 per test. The laboratory used by the University during the study period charged $85.00 per test. This fee included testing supplies and a courier to the laboratory. This price was used as a representative charge for PCR-based testing in cost analysis. If a PCR-based testing strategy for asymptomatic screening in the athletics program were used, it would have cost the University $284,920.00 to run the 3352 tests. At a cost of $20.13 per test, the University spent only $67,475.76 to test all 3352 specimens and $1785.00 to perform confirmatory PCR tests on the 21 positive cases. This resulted in cost savings to the University of $215,659.24 by employing the antigen-first strategy.

The asymptomatic testing program was not intended to provide data to calculate the performance characteristics of the BD Veritor test. Real-world data was interpreted to answer a real-world question. As a result, no collection of data was needed to calculate sensitivity, specificity, or NPV. In order to make these calculations, PCR tests would have to be performed on all subjects regardless of their antigen test results. Unfortunately, this approach was not logistically or economically feasible. Accordingly, the rate of false-negative test results yielded by the BD Veritor system during this study is unknown. However, owing to the NPVs published for the Abbott and Sofia tests and the fact that most athletic personnel were tested multiple times a week, the belief is that the number of false-negative tests was low.^8^^,^^10^

During the experience with the BD Veritor tests, cassettes were read using an analyzer to avoid human error. Although the analyzer was used as the standard to determine a positive or negative result, those using the analyzer would also do a visual check on each cassette. Two red lines on the cassette indicate that the test is positive. Users noted that cassettes without two visible red lines in the test window that were read as positive by the analyzer were often determined to be false positives upon confirmatory PCR testing. This seemed to be the case with the individuals who had multiple false-positive results. Although the tests are not intended to be read visually, this observation led to question how prone the analyzer was to this type of reading error. Unfortunately, tracking to see if this phenomenon was more prone to occur with any specific analyzer was not performed.

Overall, the belief is that a 2-stage testing approach using the BD Veritor System for screening asymptomatic individuals for SARS-CoV-2 is efficient and economical. Even with the low PPV noted for the antigen test used in this approach, the absolute number of individuals that required confirmatory testing with a PCR test was 21 out of 3352 (1% of the tests performed). The timeliness and cost-benefits realized using the BD Veritor System antigen-based testing outweighed the low PPV.

## 5 Conclusion

Although the BD Veritor System SARS-CoV-2 antigen tests were found to have a low PPV among the asymptomatic population, combination with reflex PCR testing of positive results provides an effective and sustainable means to provide screening for University athletes, coaches, and staff.

## Funding

Research equipment was funded and supported by Ferris State University.

## Declaration of Competing Interest

The authors declare that they have no known competing financial interests or personal relationships that could have appeared to influence the work reported in this paper.

The authors declare the following financial interests/personal relationships which may be considered as potential competing interests:

Casey Kepczynski, Jaelin Genigeski, Daniel deRegnier, Emmanuel Jadhav, Minji Sohn, Michael Klepser.
